# Advances in the Core Role and Mechanisms of Mitochondrial Dysfunction in Alzheimer's Disease

**DOI:** 10.1002/brb3.71418

**Published:** 2026-07-08

**Authors:** Tianyi Gu, Hangyan Guo, Zixin Guo, Shengyu Hua

**Affiliations:** ^1^ College of Traditional Chinese Medicine Tianjin University of Traditional Chinese Medicine Tianjin China

**Keywords:** Alzheimer's disease, inflammation, mitochondrial dysfunction, mitophagy, neurodegeneration

## Abstract

**Introduction:**

Alzheimer's disease (AD) is a complex neurodegenerative disorder whose pathogenesis involves multi‐level pathological alterations. This review aims to systematically elucidate the central role and multifaceted molecular mechanisms of mitochondrial dysfunction in the progression of AD.

**Methods:**

A comprehensive analysis of the existing literature was conducted, synthesizing findings from studies investigating mitochondrial involvement in AD pathology. The review focused on key mechanistic pathways, including energy metabolism deficits, oxidative stress, synaptic damage, mitochondrial dynamics, mitochondria‐associated membranes (MAMs), mitophagy, and the gut–brain axis.

**Results:**

The analysis revealed several critical mechanisms linking mitochondrial dysfunction to AD progression: (i) impaired mitochondrial energy metabolism, which establishes a causal relationship with oxidative stress and synaptic injury; (ii) dysregulation of mitochondrial fusion/fission dynamics, particularly the aberrant interactions of amyloid‐beta (Aβ) and p‐Tau with the fission protein Drp1 and the channel protein VDAC1; (iii) dysfunction of mitochondria‐associated membranes (MAMs); (iv) defective mitophagy involving both the PINK1/Parkin pathway and receptor‐mediated pathways; and (v) bidirectional crosstalk between mitochondria and the gut–brain axis. These interconnected pathways converge to amplify neuroinflammation and neuronal death.

**Conclusion:**

Accumulated evidence positions mitochondrial dysfunction as a critical hub that integrates Aβ/Tau pathology, neuroinflammation, and neuronal loss, thereby perpetuating a self‐sustaining vicious cycle in AD. Targeting mitochondrial bioenergetics, dynamics, quality control, and the mitochondria–inflammation axis offers substantial therapeutic promise. Emerging small molecules such as SS31 and DDQ have demonstrated protective effects in preclinical models. Future investigations should prioritize mechanistic dissection and translational research to facilitate the clinical development of mitochondria‐targeted therapies for AD.

## Graphical abstract

In Alzheimer's disease, Aβ and Tau trigger mitochondrial dysfunction, driving a pathological cascade that results in cognitive decline. Gut microbiota dysbiosis exacerbates this via the gut–brain axis, making mitochondria a key therapeutic target.

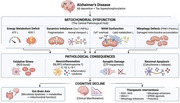

## 1 Introduction

Alzheimer's disease (AD), as the most prevalent neurodegenerative disorder, is primarily characterized by progressive cognitive decline and memory impairment, severely affecting patients' quality of life and imposing substantial socioeconomic burdens. The classic pathological hallmarks of AD include cerebral amyloid‐beta (Aβ) deposition forming amyloid plaques and neurofibrillary tangles (NFTs), the latter mainly composed of hyperphosphorylated Tau protein. These pathological alterations are considered crucial factors leading to neuronal dysfunction and loss, consequently triggering cognitive impairment and behavioral abnormalities (Roda et al. 2022; Polis and Samson 2020). However, clinical studies have demonstrated a weak correlation between Aβ plaque burden and the severity of cognitive decline, whereas Tau protein‐mediated NFTs show stronger associations with neuronal loss and clinical symptoms, suggesting Tau may serve as the primary effector molecule in neurodegeneration (Roda et al. 2022). Furthermore, complex interactions exist between Aβ and Tau proteins, collectively influencing the transcriptional regulation of synaptic function‐related genes. Downregulation of Tau expression can partially reverse Aβ‐induced transcriptional abnormalities, indicating synergistic effects between these two pathological proteins in AD pathogenesis (Roda et al. 2022). Nevertheless, the scientific community has proposed the “dual‐pathway hypothesis,” suggesting that common upstream factors simultaneously trigger both Aβ and Tau pathologies, with the immune system playing a particularly important role. Genetic factors such as the apolipoprotein E (APOE) ε4 allele are considered crucial bridges connecting Aβ and Tau pathology (Roda et al. 2022; X. Guo et al. 2025).

In recent years, with in‐depth investigation into AD pathological mechanisms, mitochondrial dysfunction has been recognized as a crucial intrinsic mechanism in AD pathogenesis. Mitochondria serve not only as the central hub of cellular energy metabolism, responsible for generating the majority of ATP, but also participate in regulating various cellular processes including oxidative stress responses, calcium ion homeostasis, and apoptosis. Particularly, the high energy dependence of neurons makes them exceptionally vulnerable to mitochondrial functional abnormalities, constituting a key factor in their selective vulnerability (F. Yang et al. 2025; Sharma et al. 2021; Monsalvo‐Maraver et al. 2023). Mitochondrial dysfunction leads to impaired energy metabolism and promotes excessive generation of reactive oxygen species (ROS), consequently inducing oxidative stress that damages cell membranes, proteins, and DNA, thereby exacerbating neuronal injury and death (Timalsina et al. 2025; Spina et al. 2025). Furthermore, disturbances in mitochondrial maintenance of calcium homeostasis have been demonstrated to closely correlate with neuronal dysfunction and apoptosis in AD (Garcia‐Casas et al. 2023). These mitochondrial‐related pathological alterations not only promote abnormal deposition of Aβ and Tau proteins but also accelerate neurodegenerative processes through activation of neuroinflammatory responses (F. Yang et al. 2025; P. Qin et al. 2024).

In terms of mechanistic research, mitochondria‐associated membranes (MAMs), serving as critical platforms for intracellular calcium signaling and lipid metabolism, have been demonstrated to participate in AD pathogenesis through their functional abnormalities. MAMs regulate mitochondrial dynamics, energy metabolism, autophagy, and apoptosis; their dysfunction may lead to calcium homeostasis disruption and mitochondrial stress responses, thereby promoting neuronal pathology (Proulx et al. 2021). Furthermore, mitochondrial dynamics—encompassing the processes of fission and fusion—play vital roles in maintaining mitochondrial health and function. Imbalanced expression of mitochondrial dynamics‐related proteins in AD brain tissue results in abnormal mitochondrial morphology and functional impairment (F. Yang et al. 2025; Monsalvo‐Maraver et al. 2023). The autophagy mechanism, particularly mitophagy—the selective clearance of damaged mitochondria—plays a central role in maintaining neuronal mitochondrial quality control. Disruption of this process exacerbates the accumulation of damaged mitochondria and neuroinflammatory responses (McGill Percy et al. 2025; Butler et al. 2022). Additionally, mitochondrial‐mediated inflammatory responses, such as the activation of the NLRP3 inflammasome and subsequent release of proinflammatory cytokines, have emerged as significant mechanisms underlying chronic neuroinflammation in AD (P. Qin et al. 2024).

**FIGURE 1:**
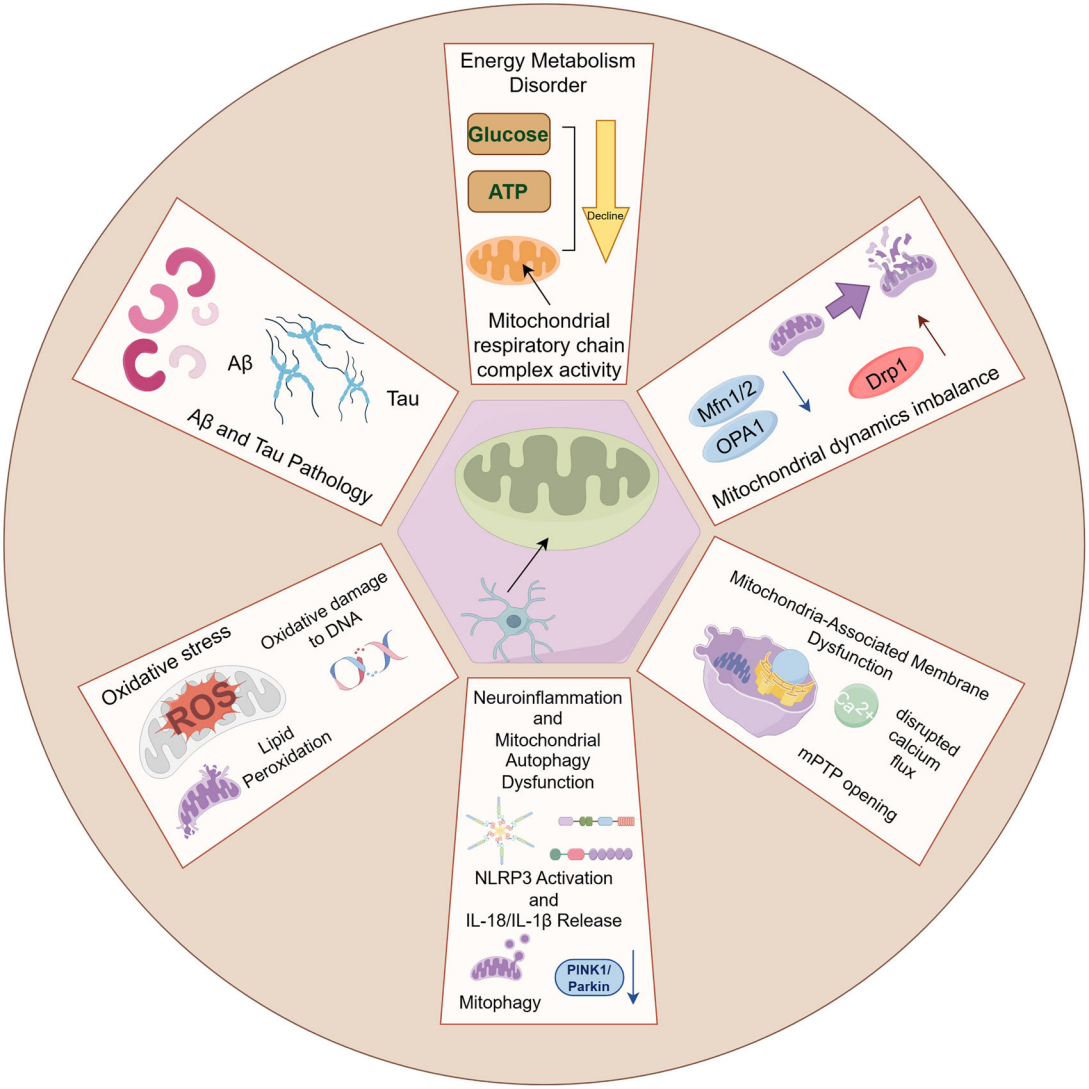
Schematic diagram of the core pathological network and vicious cycle of mitochondrial dysfunction in Alzheimer's disease (AD). In AD, Aβ deposition and abnormal aggregation of Tau protein, as core pathological proteins, can induce mitochondrial energy metabolism disorders, dynamic imbalance, and mitochondrial‐associated membrane (MAM) dysfunction (Yu et al. 2021). Energy metabolism disorders lead to excessive generation of reactive oxygen species (ROS), triggering oxidative damage and activating neuroinflammation centered around the NLRP3 inflammasome (Litwiniuk et al. 2021). Mitochondrial dynamic imbalance further impairs mitophagy and induces the opening of the mitochondrial permeability transition pore (mPTP), while MAM dysfunction mediates calcium overload, ultimately activating neuronal apoptosis pathways (Z. Zhang et al. 2024). The activated inflammatory response, in turn, exacerbates mitochondrial damage, forming a self‐perpetuating vicious cycle that drives neuronal loss and cognitive decline.The Fig 1 was created by Figdraw. Copyright Code: YIUOId665f.

Thus, research on AD pathogenesis has expanded beyond the traditional focus on Aβ and Tau pathology to include in‐depth exploration of mitochondrial dysfunction and its associated cellular mechanisms. Mitochondrial dysfunction not only constitutes a significant component of early pathological changes in AD but also provides novel perspectives and potential targets for understanding disease initiation and progression (F. Yang et al. 2025; Sharma et al. 2021; Spina et al. 2025). Based on this understanding, mitochondrial‐targeted intervention strategies have garnered increasing attention in recent years. These approaches—including improving mitochondrial energy metabolism, counteracting oxidative stress, regulating mitochondrial dynamics, and promoting mitophagy—offer new hope for AD treatment (McGill Percy et al. 2025; Złotek et al. 2023; Almikhlafi et al. 2023). This article will systematically review the role of mitochondrial dysfunction in AD neurodegeneration and examine potential intervention strategies, integrating the latest research advances to explore future therapeutic directions. The core pathological network and vicious cycle involving these mechanisms are summarized in **Figure** **1**.

## 2 Mitochondrial Dysfunction and Molecular Mechanisms in AD

### 2.1 Mitochondrial Energy Metabolism Impairment and Neuronal Damage

Mitochondria, as the energy powerhouses of cells, play a central role in neuronal damage in AD. In the brain tissue of AD patients, significantly reduced activity of mitochondrial respiratory chain complexes leads to decreased ATP production, directly impairing neuronal energy supply. This energy deficit not only restricts normal physiological functions of neurons but also heightens their susceptibility to various stressors, thereby exacerbating neuronal damage and apoptosis. For instance, studies have demonstrated that reduced mitochondrial proteins in AD brain tissue correlate with depletion of total glutathione (GSH), whose synthesis consumes ATP—indicating that energy deficiency constitutes a critical limiting factor for antioxidant defense (Alves et al. 2025). The seminal work from the Reddy laboratory established the central role of mitochondrial dysfunction in the pathogenesis of AD. As early as 2006, Reddy and colleagues systematically articulated the mechanism by which amyloid precursor protein (APP) derivatives induce mitochondrial oxidative damage through the generation of free radicals (Reddy 2006). Their study revealed that in the brains of transgenic mice expressing mutant APP (Tg2576 line), the expression of mitochondria‐associated genes was upregulated as early as 2 months of age, suggesting that mitochondrial dysfunction represents a very early cellular event in the pathological progression of AD (Reddy et al. 2004).

Furthermore, energy metabolism imbalance resulting from mitochondrial dysfunction induces oxidative stress responses, increases ROS generation, and further damages mitochondrial and cellular structures, creating a vicious cycle that promotes neuronal apoptosis. For example, abnormal accumulation of Aβ and Tau proteins can disrupt mitochondrial inner membrane potential and respiratory chain function, inducing elevated ROS levels and cellular energy metabolism impairment (Stelmashchuk et al. 2025; Z. Huang et al. 2020). The causal chain from“mitochondrial respiratory chain impairment” to “synaptic plasticity deficits” can be delineated as follows: Mitochondrial respiratory chain dysfunction leads to a reduction in ATP production and an explosion of ROS, which in turn triggers lipid peroxidation, protein oxidation, and DNA damage. This cascade creates an energy crisis in the highly energy‐dependent presynaptic terminals, resulting in vesicle cycling disorders. Simultaneously, oxidative stress and cellular damage cause dendritic spine loss in the postsynaptic region. Consequently, long‐term potentiation (LTP) is impaired, ultimately leading to a decline in synaptic plasticity (Todorova and Blokland 2017; Z. Li et al. 2004; Glaser et al. 2010).

Within this established framework, the recently identified protein OCIAD1 offers a significant extension. Research indicates that OCIAD1 acts as a critical pathogenic factor downstream of the Aβ/GSK‐3β signaling pathway; its dysregulation or functional impairment leads to impaired mitochondrial biogenesis and reduced activity of respiratory chain complexes, thereby exacerbating neuronal metabolic stress and structural damage (R. Liu and Zhou 2025). This mechanism reveals OCIAD1 as a critical node connecting Aβ toxicity with mitochondrial energy metabolism deficits, positioning it as a potential therapeutic target.

Furthermore, mitochondrial metabolic impairment promotes oxidative stress responses, thereby inducing neuronal damage and apoptosis. Under conditions of energy metabolism dysfunction, the decline in mitochondrial membrane potential triggers the opening of the mitochondrial permeability transition pore (mPTP), leading to the release of pro‐apoptotic factors such as cytochrome c, which activates the apoptotic cascade (Olatona et al. 2025). Additionally, excessive ROS resulting from mitochondrial dysfunction induces lipid peroxidation and protein oxidation, damaging cell membranes and enzyme activity, thereby further exacerbating neuronal injury (Ali et al. 2024). Oxidative stress can also activate inflammatory signaling pathways, inducing neuroinflammatory responses and forming a vicious cycle that promotes sustained neuronal damage (Y. Mishra et al. 2025). Consequently, through the dual pathways of reduced energy metabolism and increased oxidative stress, mitochondrial dysfunction serves as a core mechanism underlying neuronal injury in AD.

Intervention strategies targeting mitochondrial energy metabolism deficits are gaining increasing attention. For instance, the SIRT1 activator ginsenoside Rc promotes mitochondrial biogenesis, enhances electron transport chain complex activity, and increases ATP production, thereby improving neuronal metabolic function and survival rates (Q. Huang et al. 2021). Concurrently, modulation of signaling pathways such as AMPK and PGC‐1α helps maintain mitochondrial homeostasis and autophagy, supporting mitochondrial function and neuronal stability (Z. Liu et al. 2025). Furthermore, natural compounds including sulforaphane and rutin demonstrate potential for mitigating AD‐related neuronal damage through antioxidant effects and restoration of mitochondrial function (Y. Chen et al. 2024; Kandy et al. 2022).

In summary, impaired mitochondrial respiratory chain function in the brains of AD patients leads to reduced ATP production, severely compromising neuronal energy supply. The OCIAD1 protein‐mediated mitochondrial dysfunction serves as a critical pathogenic factor downstream of the Aβ/GSK‐3β signaling pathway, promoting neuronal vulnerability and synaptic damage. Mitochondrial metabolic deficits exacerbate oxidative stress responses, inducing neuronal injury and apoptosis, thereby forming a vicious cycle. Therapeutic strategies targeting mitochondrial energy metabolism impairments—such as activation of the SIRT1/PGC‐1α signaling pathway and enhancement of mitophagy and mitochondrial biogenesis—provide novel insights and potential therapeutic targets for AD treatment (Alves et al. 2025; Olatona et al. 2025; Q. Huang et al. 2021; Z. Liu et al. 2025).

### 2.2 Mitochondrial Dynamic Imbalance: Dysregulation of Fusion and Fission

Mitochondrial homeostasis refers to the process by which mitochondria maintain their morphological and functional equilibrium through continuous fusion and fission. This dynamic process is critical for cellular energy metabolism, redox homeostasis, and cell survival. Fusion is primarily mediated by outer membrane fusion proteins MFN1 and MFN2, along with inner membrane fusion protein OPA1, while fission depends on regulatory factors such as DRP1, FIS1, and MFF. Under physiological conditions, the balance between fusion and fission maintains the integrity and functionality of the mitochondrial network.

In the pathological progression of AD, abnormal accumulation of Aβ significantly disrupts the balance of mitochondrial dynamics, evidenced by increased expression of fission genes Drp1 and Fis1 and decreased expression of fusion genes Mfn1, Mfn2, and Opa1, which leads to increased mitochondrial fragmentation, structural damage, and functional defects. This dysregulation of mitochondrial fusion and fission not only impairs the axonal transport of mitochondria, preventing their proper distribution to synapses, but also directly triggers synaptic degeneration and neuronal damage, highlighting the critical role of imbalanced mitochondrial dynamics in AD neurodegeneration (Reddy et al. 2012).

Studies have revealed significantly reduced expression of fusion proteins MFN1, MFN2, and OPA1, along with elevated activity and expression levels of the fission protein DRP1 in both AD brain tissue and cellular models. This imbalance promotes excessive mitochondrial division (Garcia et al. 2022; Y. ‐R. Qin et al. 2022). The pivotal study by Wang and colleagues provided the first confirmation of this imbalance in brain tissue from AD patients. Their findings revealed significantly reduced levels of DRP1, OPA1, Mfn1, and Mfn2, alongside a marked increase in Fis1 levels. More importantly, the distribution of these proteins within neurons was altered, shifting from a uniform presence throughout the neuron to clustering within the cell body, with a concomitant marked reduction within neurites, indicating that mitochondrial distribution is also abnormal (X. Wang et al. 2009).

**FIGURE 2:**
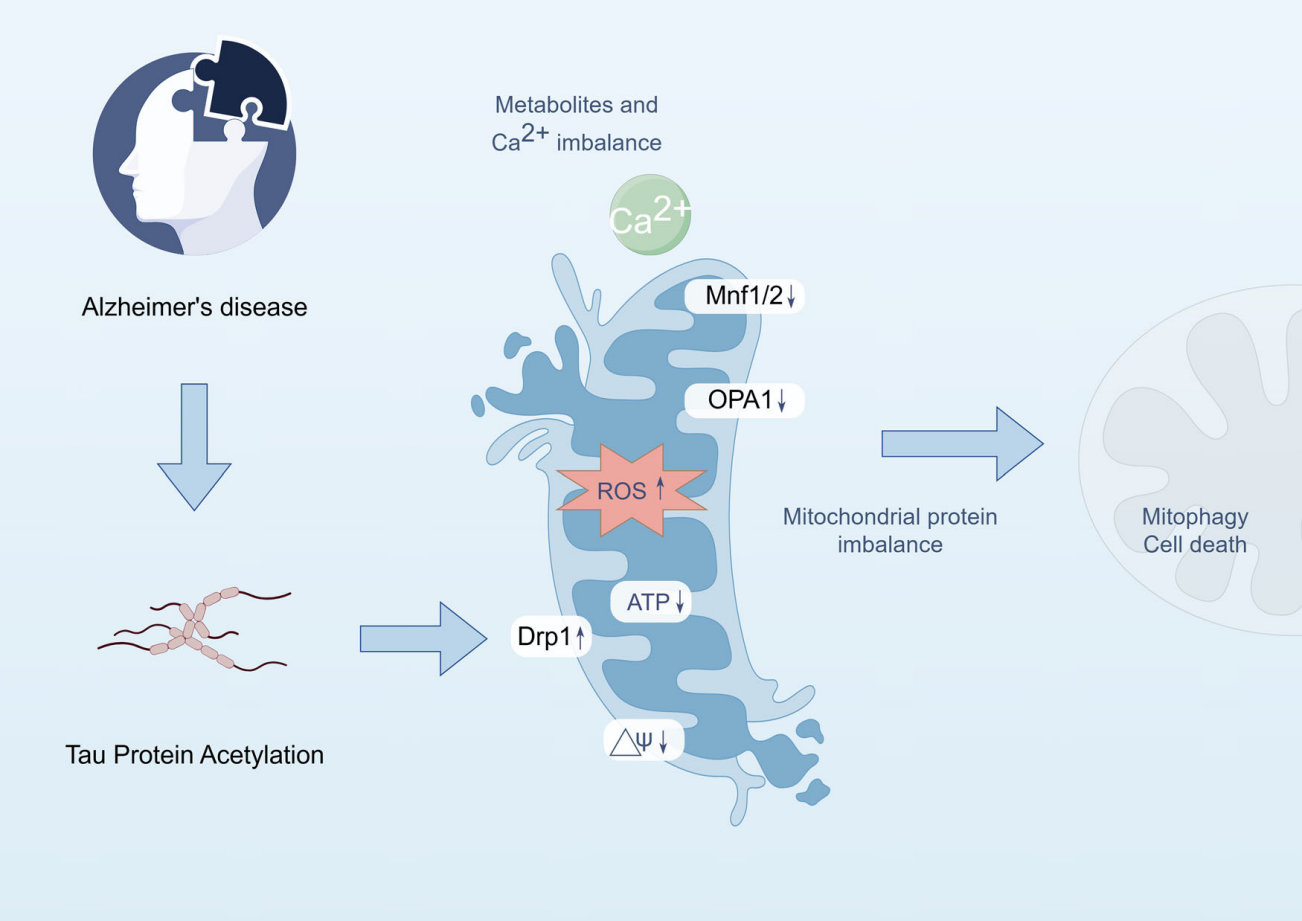
Schematic diagram of Tau acetylation‐mediated mitochondrial dynamic imbalance and neuronal death pathways. In the pathological progression of AD, acetylation modification of the Tau protein, as a key upstream event, abnormally activates the mitochondrial fission protein DRP1, thereby driving excessive mitochondrial fission. Meanwhile, the downregulation of fusion proteins (such as MFN1/2 and OPA1) results in the inability to effectively repair and fuse the mitochondrial fragments produced by fission. The persistent accumulation of these fragmented mitochondria leads to a decrease in mitochondrial membrane potential, burst generation of ROS, and a crisis in ATP synthesis, ultimately activating the intrinsic apoptosis pathway and causing neuronal death (Reddy et al. 2012). This constitutes a core pathological axis from post‐translational protein modification to organelle dysfunction, culminating in neurodegenerative outcomes. The Fig 2 was created by Figdraw. Copyright Code: YPTYY224e2.

How, then, does AD pathology drive this excessive fission? Latest studies have once again confirmed that Aβ and p‐Tau are key biomarkers for AD diagnosis and prediction (Pradeepkiran and Reddy 2020). Research has revealed that Aβ and p‐Tau can directly engage in aberrant interactions with DRP1, enhancing its GTPase enzymatic activity and consequently driving excessive mitochondrial fragmentation (Manczak et al. 2011). Furthermore, Tau protein acetylation exacerbates the fragmented state of mitochondria by reducing fusion protein levels, thereby aggravating mitochondrial dysfunction and neuronal apoptosis (J. ‐F. Zhang et al. 2024). This process, in which Tau acetylation drives excessive mitochondrial fission and downstream neuronal injury, is illustrated in Figure 2.

Mitochondrial dynamic imbalance‐induced functional impairments are primarily manifested through mitochondrial membrane potential depolarization, reduced respiratory chain complex activity, decreased ATP production, and increased generation of ROS. These abnormalities lead to neuronal energy metabolism dysregulation, triggering apoptotic pathways and accelerating the progression of neurodegenerative pathology (Green et al. 2022; Grel et al. 2023). Concurrently, disrupted mitochondrial dynamics compromise mitochondrial DNA (mtDNA) integrity and diminish mitochondrial quality control capacity, further exacerbating mitochondrial damage (Wisniewski et al. 2024).

In recent years, research has focused on restoring mitochondrial dynamic balance as a therapeutic approach to mitigate AD‐related mitochondrial dysfunction. For instance, either inhibiting the activity of the fission protein DRP1 or promoting the expression of fusion proteins MFN1/2 can partially restore mitochondrial morphology, enhance respiratory function, reduce ROS levels, and delay neuronal injury (Garcia et al. 2022; Y.‐R. Qin et al. 2022). Specific small molecules such as Mdivi‐1 (a DRP1 inhibitor) and drugs like artesunate have demonstrated potential in modulating mitochondrial dynamics and alleviating neuronal damage in AD models (Y.‐R. Qin et al. 2022). Furthermore, exercise intervention has been shown to upregulate MFN2 expression, improve mitochondrial fusion, and enhance mitochondrial function, thereby attenuating AD pathological progression (H. Li 2025).

In conclusion, the imbalance between mitochondrial fusion and fission represents a crucial mechanism underlying neuronal metabolic dysregulation and cell death in AD. This disruption not only compromises mitochondrial structural integrity but also leads to functional impairment. Thus, restoring mitochondrial dynamic homeostasis through modulation of relevant fusion and fission protein expression and activity has emerged as a potential therapeutic strategy for AD. Further investigation in this direction will provide both theoretical foundations and experimental evidence for elucidating AD pathological mechanisms and developing novel intervention approaches.

### 2.3 Abnormal Interactions Between Aβ/p‐Tau and Drp1, Voltage‐Dependent Anion Channel 1 (VDAC1)

Beyond directly impairing mitochondrial structure, Aβ and p‐Tau can drive excessive mitochondrial fission and dysfunction through aberrant interactions with Drp1, a key regulator of mitochondrial dynamics. Research indicates that Drp1 physically interacts with Aβ, and this interaction intensifies with the progression of AD (Manczak et al. 2016). This abnormal binding directly leads to elevated GTPase activity of Drp1, resulting in excessive mitochondrial fragmentation. Similarly, p‐Tau has been shown to interact aberrantly with Drp1, which similarly enhances Drp1 enzymatic activity and disrupts mitochondrial dynamics homeostasis (Kandimalla et al. 2016).

Genetic intervention experiments further confirm the central role of Drp1 in this toxic cascade. By crossing Drp1 heterozygous knockout mice (Drp1±) with AD model mice, studies demonstrate that partial reduction of Drp1 levels effectively blocks pathological damage. In APP transgenic mice, partial Drp1 deficiency significantly reduces soluble Aβ levels, restores the expression of mitochondrial fusion proteins and biogenesis‐related genes, and improves synaptic activity (Manczak et al. 2016). Likewise, in Tau transgenic mice, reduced Drp1 expression not only reverses Tau‐induced abnormalities in mitochondrial dynamics and dysfunction but also markedly decreases phosphorylated Tau levels, thereby alleviating synaptic toxicity (Kandimalla et al. 2016). This evidence establishes Drp1 as a critical node in the amplification of Aβ and p‐Tau toxicity, suggesting that targeting Drp1 may hold therapeutic potential for AD and related tauopathies.

Beyond their interaction with the dynamics protein Drp1, Aβ and p‐Tau further exacerbate neuronal damage through aberrant interactions with VDAC1, a key channel protein on the mitochondrial outer membrane. As the “gatekeeper” of mitochondrial metabolism, VDAC1 not only regulates the transmembrane transport of ions and metabolites but also serves as a critical node in mitochondria‐mediated apoptosis. Research has revealed significantly elevated VDAC1 expression levels in post‐mortem brain tissues from AD patients and in APP transgenic mice, where such overexpression is sufficient to trigger cell death, implying a significant role for VDAC1 in neuronal loss (Shoshan‐Barmatz et al. 2018).

At the molecular mechanistic level, both Aβ and p‐Tau can directly bind to VDAC1. Aβ interacts with the N‐terminal domain of VDAC1 via its GXXXG motif, a binding that not only promotes VDAC1 oligomerization but also leads to cytochrome c release, ultimately initiating mitochondria‐mediated apoptosis. Concurrently, the aberrant interaction between p‐Tau and VDAC1 blocks mitochondrial pores, interrupting the normal flux of metabolites, leading to mitochondrial dysfunction and neuronal damage. Furthermore, genetic intervention studies have provided causal evidence: crossing VDAC1 heterozygous knockout mice (VDAC1±) with Tau transgenic mice resulted in double mutant mice exhibiting a significant decrease in mitochondrial number and an increase in mitochondrial length in the hippocampus and cortex, indicating an effective reversal of excessive mitochondrial fragmentation. This reduction in VDAC1 levels not only restored synaptic protein expression and dendritic spine density but also activated PINK1‐Parkin‐mediated mitophagy, thereby clearing damaged mitochondria and ameliorating behavioral deficits. These findings establish VDAC1 as a significant amplifier of Aβ and p‐Tau toxic effects in AD pathology, suggesting that inhibiting the aberrant function or overexpression of VDAC1 may represent a novel therapeutic strategy for AD (Vijayan et al. 2022).

### 2.4 Mitochondria‐Associated Membrane Dysfunction

Mitochondria‐associated membranes (MAMs) not only serve as platforms for interactions between Aβ, p‐Tau, and proteins such as VDAC1, but their own dysfunction also plays a critical role in AD pathology. Functioning as specialized contact sites between mitochondria and the endoplasmic reticulum (ER), MAMs structurally connect these two organelles and play a key role in calcium homeostasis, lipid metabolism, autophagy, and signal transduction. These membrane domains are enriched with various protein complexes—such as inositol 1,4,5‐trisphosphate receptors (IP3R), glucose‐regulated protein 75 (GRP75), and voltage‐dependent anion‐selective channel 1 (VDAC1)—that collectively mediate calcium transfer between the ER and mitochondria and coordinate diverse metabolic activities (Truong et al. 2025; Eysert et al. 2020). In AD, MAMs dysfunction becomes particularly prominent, characterized by shortened ER–mitochondria contact distances that result in disrupted calcium flux. This dysregulation subsequently triggers the opening of the mPTP, ultimately inducing neuronal apoptosis.

In AD patient brain tissue and cellular models, the spatial architecture of MAMs undergoes significant alterations, characterized by increased ER–mitochondria contact area accompanied by functional disturbances. These pathological changes are closely associated with MAMs dysfunction, including dysregulated calcium signaling, disrupted lipid metabolism, and impaired autophagy (Yu et al. 2021; Fernandes et al. 2021). MAMs impairment not only leads to compromised mitochondrial energy metabolism, ROS accumulation, and activation of cellular stress responses but also promotes abnormal accumulation of AD‐related proteins. Notably, the aberrant enrichment of APP and its metabolic product C99 within MAMs further exacerbates both mitochondrial and ER dysfunction (Eysert et al. 2020; Pera et al. 2020).

Furthermore, the structural integrity of MAMs is closely linked to AD‐associated genetic mutations. For instance, mutations in APP and PSEN genes—particularly those observed in familial AD cases—compromise the stability and function of MAMs protein complexes, ultimately leading to mitochondrial calcium overload and disrupted lipid metabolism (Yu et al. 2021; Eysert et al. 2020). Notably, MAMs pathological alterations are not restricted to AD but are also prevalent in other neurodegenerative disorders, indicating that MAMs dysfunction represents a fundamental molecular basis for neurodegeneration (Johri and Chandra 2021; Raeisossadati and Ferrari 2022). Consequently, restoring MAMs architecture and function, along with recalibrating calcium homeostasis and lipid metabolism, has emerged as a promising therapeutic direction for AD and related neurodegenerative conditions (Truong et al. 2025; Z. Li et al. 2023).

It can be concluded that MAMs, as critical membrane structures connecting mitochondria and the ER, serve as hubs for cellular calcium signaling, lipid metabolism, and autophagy regulation. Their dysfunction contributes to AD pathogenesis by inducing mitochondrial calcium overload, mPTP activation, and cellular apoptosis. Mutations in AD‐associated genes further exacerbate MAMs abnormalities, revealing the central role of MAMs in neurodegeneration and providing a theoretical foundation and novel therapeutic perspectives for MAMs‐targeted interventions (Yu et al. 2021; Eysert et al. 2020; Zellmer et al. 2025).

#### 2.4.1 Mitochondria‐Mediated Oxidative Stress and Neuroinflammatory Response

#### 2.4.2 The Role of Mitochondrial Oxidative Stress in AD

Mitochondria, as the primary cellular source of ROS, play a central role in AD. In AD brain tissue, mitochondrial dysfunction represents an early pathological event, characterized predominantly by excessive ROS production. This subsequently induces oxidative damage to mitochondrial DNA (mtDNA), proteins, and lipids, establishing a vicious cycle that further exacerbates mitochondrial impairment and cellular damage (P. Yang et al. 2020; Ademowo et al. 2024). Given the particular vulnerability of neurons to oxidative damage, mitochondria‐derived ROS directly contributes to neuronal metabolic imbalance, energy deficiency, and apoptosis, thereby accelerating both the initiation and progression of AD (Gupta et al. 2025; Epremyan et al. 2022).

Excessive ROS generation not only disrupts mitochondrial structure and function but also exacerbates protein misfolding and aggregation, particularly the pathological alterations of Aβ and Tau proteins. Oxidative stress promotes Tau oligomerization and hyperphosphorylation, aTccelerating NFT formation, while simultaneously enhancing Aβ aggregation under oxidative conditions to form pathogenic plaques (Du et al. 2022; Bhatia and Sharma 2021). Furthermore, abnormal ROS accumulation activates inflammatory responses and induces mitochondrial membrane potential dissipation, promoting the activation of apoptotic pathways and resulting in progressive neuronal damage (L. Wang et al. 2025; Shah et al. 2025).

Furthermore, mitochondrial abnormalities disrupt interneuronal energy metabolism and calcium homeostasis, impairing synaptic function and ultimately leading to cognitive dysfunction (Verma et al. 2023; S. ‐Y. Chen et al. 2020). Studies demonstrate a bidirectional feedback mechanism between mitochondrial oxidative stress and AD pathology, where ROS serves as both a cause and consequence of mitochondrial dysfunction, establishing a self‐perpetuating vicious cycle (Ademowo et al. 2024; Awasthi et al. 2021).

Mitochondria‐targeted interventions against oxidative stress are increasingly emerging as a focal point in AD therapeutic research. Several studies have developed specific mitochondrial antioxidants, such as triphenylphosphonium (TPP)‐modified nanoparticles and the cell‐penetrating mitochondria‐targeted peptide SS‐31. These compounds effectively scavenge mitochondrial ROS, stabilize mitochondrial dynamics, and attenuate Aβ deposition and abnormal Tau phosphorylation, consequently improving cognitive function (P. Yang et al. 2020; L. Wang et al. 2025; Calvo‐Rodriguez et al. 2024). Furthermore, natural compounds including quercetin and tangeretin demonstrate potential neuroprotective effects through their capacity to modulate oxidative stress and restore mitochondrial function (Adnan et al. 2025; Y. He et al. 2025).

Excessive production of ROS induces mitochondrial DNA and protein damage, triggers protein misfolding and aggregation, and promotes the progression of both Aβ and Tau pathology, ultimately leading to neuronal dysfunction and cognitive decline. Consequently, mitochondrial oxidative stress holds a pivotal position in the pathogenesis of AD, and therapeutic strategies targeting this mechanism present broad application prospects, representing a crucial direction for both AD research and clinical intervention (P. Yang et al. 2020; L. Wang et al. 2025; Awasthi et al. 2021).

#### 2.4.3 Interaction Between Mitochondria and the NLRP3 Inflammasome

Mitochondrial dysfunction serves as a critical driver for NLRP3 inflammasome activation. Within cells, mitochondria not only play a central role in energy supply but also participate in regulating redox status, calcium homeostasis, and apoptosis, among other vital biological processes. Mitochondrial damage leads to the release of danger signaling molecules such as mitochondrial DNA (mtDNA) and ROS, which can directly or indirectly activate the NLRP3 inflammasome, thereby triggering inflammatory responses. For example, the oxidized form of mtDNA (ox‐mtDNA), when released into the cytoplasm, can directly bind to NLRP3, promoting its assembly and activation and subsequently enhancing the production of inflammatory mediators IL‐1β and IL‐18 (Qiu et al. 2022; B. Huang et al. 2024). ROS, as hallmark markers of mitochondrial damage, function both as intracellular signaling molecules and key activators of the NLRP3 inflammasome, facilitating the assembly and activation of the NLRP3 complex through oxidative stress mechanisms (Holley and Schroder 2020; T. Zhang et al. 2021).

Furthermore, mitochondrial dynamics—encompassing the processes of fission and fusion—play an essential regulatory role in NLRP3 inflammasome activity. Imbalances in mitochondrial dynamics lead to decreased mitochondrial membrane potential, facilitating the release of mtDNA and ROS, which subsequently activates NLRP3 (S. R. Mishra et al. 2021; Bai et al. 2024). Mitophagy, as a crucial mechanism for maintaining mitochondrial quality control, restricts excessive NLRP3 activation by clearing damaged mitochondria and alleviating inflammatory responses. Conversely, impaired mitophagy exacerbates hyperactivation of the inflammasome (Wu and Cheng 2022; Wu et al. 2021).

Studies have further revealed that mitochondria provide essential spatial platforms for the assembly and activation of the NLRP3 inflammasome. The NLRP3 protein is initially recruited to the mitochondrial surface before translocating to the Golgi apparatus to complete inflammasome assembly (Luo et al. 2024; Arumugam et al. 2022). Mitochondria‐associated proteins such as MARCH5 facilitate the formation of the NLRP3‐NEK7 complex by regulating NLRP3 ubiquitination, thereby enhancing inflammasome activation (Park et al. 2023). Additionally, the oligomerization process of the mitochondrial voltage‐dependent anion channel (VDAC) has been demonstrated to be closely associated with NLRP3 assembly. VDAC serves as a permeability conduit for macromolecules including mtDNA and proteins, promoting inflammasome activation (Baik et al. 2023; Ma et al. 2024).

Conversely, the inflammatory response itself can reciprocally impair mitochondrial function, establishing a maladaptive positive feedback loop. Activated NLRP3 inflammasomes trigger substantial release of downstream inflammatory cytokines such as IL‐1β and IL‐18. These cytokines induce intracellular oxidative stress and mitochondrial damage, leading to loss of mitochondrial membrane potential, increased ROS production, and additional mtDNA release, which in turn further activates the NLRP3 inflammasome, creating a self‐perpetuating vicious cycle (Ayyubova and Madhu 2025; Yan et al. 2024). This positive feedback mechanism has been confirmed in multiple neurodegenerative disorders, particularly in AD, where mitochondrial damage‐induced NLRP3 inflammasome activation exacerbates neuroinflammation, thereby accelerating neuronal injury and cognitive decline (Litwiniuk et al. 2021; Ayyubova and Madhu 2025).

On one hand, mitochondrial damage activates the NLRP3 inflammasome through the release of mtDNA and ROS, leading to the secretion of inflammatory cytokines IL‐1β and IL‐18, thereby amplifying inflammatory responses. Conversely, the inflammatory response further impairs mitochondrial function, establishing a maladaptive positive feedback loop that ultimately promotes neurodegeneration and disease progression. Therapeutic interventions targeting this circuit—such as enhancing mitophagy to restore mitochondrial function and inhibiting NLRP3 inflammasome activity—hold significant research, clinical translation, and therapeutic potential (Wu and Cheng 2022; Ayyubova and Madhu 2025; T. Zhang et al. 2022).

#### 2.4.4 Potential Therapeutic Targets for Inflammation Regulation

In the pathogenesis of AD, neuroinflammation is recognized as a critical driving factor. Multiple studies indicate that neuroinflammation not only exacerbates neuronal damage but also promotes disease progression through activation of inflammasomes such as NLRP3. Mitochondrial dysfunction serves as a pivotal element in initiating and sustaining this inflammatory response. Particularly when the mitochondrial fission regulatory protein Drp1 becomes aberrantly activated, mitochondrial homeostasis is disrupted, further potentiating NLRP3 inflammasome activation. This triggers the secretion of proinflammatory cytokines and establishes a neuroinflammation‐amyloid cascade, ultimately amplifying Aβ deposition and Tau pathology while accelerating neurodegeneration (Sbai et al. 2023). Consequently, restoring mitochondrial function and suppressing inflammatory signaling pathways have emerged as promising therapeutic strategies for AD.

G protein‐coupled receptor 40 (GPR40) agonists such as TUG469 have been demonstrated to alleviate neuroinflammation and cognitive impairment by modulating mitochondrial function and suppressing NLRP3 inflammasome activation. Mechanistically, GPR40 agonists restore mitochondrial membrane potential, reduce ROS generation, and attenuate mitochondrial‐mediated cellular stress responses and pro‐inflammatory signaling. Consequently, they inhibit excessive activation of microglia and astrocytes, decrease the secretion of inflammatory cytokines including TNF‐α, IL‐1β, and IL‐6, and ultimately improve the neural environment (Sbai et al. 2023; Tayanloo‐Beik et al. 2022). Furthermore, GPR40 agonists may promote mitochondrial homeostasis by regulating mitochondrial dynamics (fusion and fission) and enhancing mitophagy, thereby facilitating the clearance of damaged mitochondria. These mechanisms collectively contribute to the amelioration of inflammatory responses and oxidative stress, protecting neurons from damage (Song et al. 2021; X. Wang et al. 2022).

Notably, neuroinflammation in the brains of AD patients is predominantly mediated by activated microglia, where mitochondrial dysfunction‐induced overproduction of ROS serves as a key driver of microglial inflammatory responses. Concurrently, NLRP3 inflammasome activation amplifies this inflammatory cascade (Sbai et al. 2023; Agrawal and Jha 2020). By intervening in this process, GPR40 agonists show promise in restoring microglial homeostasis, breaking the vicious cycle of neuroinflammation, and potentially slowing AD pathological progression.

These findings collectively demonstrate that restoring mitochondrial function, inhibiting NLRP3 inflammasome activation, and alleviating neuroinflammation and cognitive impairment represent promising therapeutic targets for AD. Future research should focus on further elucidating the underlying mechanisms, optimizing drug design, and improving brain‐targeted delivery efficiency to facilitate clinical translation. Additionally, combining multiple intervention approaches—such as antioxidants, mitochondrial quality control regulators, and anti‐inflammatory agents—may enable more comprehensive and effective therapeutic strategies for AD (Sbai et al. 2023; Terzo et al. 2021; Koshatwar et al. 2023).

### 2.5 Regulation and Role of Mitophagy in AD

#### 2.5.1 Mitophagy Regulates Mitochondrial Quality Control

Mitophagy represents a specialized form of selective autophagy dedicated to the clearance of damaged or dysfunctional mitochondria, playing a central role in maintaining mitochondrial homeostasis and cellular energy metabolic balance (Pradeepkiran and Reddy 2020). As the primary site of cellular energy metabolism, mitochondria are responsible for ATP production and regulation of ROS. The functional integrity of mitochondria is directly essential for cellular survival and function. Damaged mitochondria not only compromise energy supply but also trigger excessive ROS release, causing oxidative stress and cellular injury. Therefore, the timely removal of these impaired mitochondria is a necessary process for maintaining cellular homeostasis.

Mitophagy executes the clearance of damaged mitochondria through a cascade of signaling pathways that recognize and encapsulate impaired organelles, subsequently delivering them to lysosomes for degradation. This process effectively prevents the detrimental effects associated with the accumulation of dysfunctional mitochondria. The canonical PINK1/Parkin‐mediated pathway represents a crucial regulatory mechanism for mitophagy: upon loss of mitochondrial membrane potential, PINK1 stabilizes and accumulates on the outer mitochondrial membrane, recruiting Parkin and promoting ubiquitination of mitochondrial proteins. This ubiquitination cascade ultimately triggers autophagosome formation and mitochondrial elimination. Furthermore, receptor‐mediated pathways involving BNIP3/NIX also contribute to the regulation of mitophagy under specific physiological contexts, underscoring the existence of diverse regulatory mechanisms (De et al. 2021; Basak and Holzbaur 2025).

In AD patients and related models, impaired mitophagy function manifests as reduced efficiency in clearing damaged mitochondria, leading to abnormal mitochondrial accumulation. This exacerbates neuronal energy metabolism deficits and oxidative stress. Such accumulation not only promotes the pathological aggregation of Aβ and Tau proteins but also induces chronic inflammatory responses and apoptosis, further aggravating neurodegenerative processes. Multiple studies have demonstrated decreased expression of PINK1 and Parkin, diminished mitophagy activity, and dysregulated related proteins such as BNIP3L/NIX in AD brain tissues (Song et al. 2021; Pradeepkiran and Reddy 2020; Kakoty et al. 2025).

Furthermore, mitophagy is closely linked to mitochondrial dynamics, as the processes of mitochondrial fission and fusion play a promotive role in initiating mitophagy. Excessive mitochondrial fission typically facilitates the segregation of damaged mitochondria, creating conditions for their subsequent selective clearance. Research has revealed that in AD and related neurodegenerative disorders, imbalanced mitochondrial dynamics—characterized by hyperactive fission and impaired fusion—hinders the normal progression of mitophagy, thereby exacerbating mitochondrial functional deficits (Xian and Liou 2021; Dorn and Dang 2022).

Multiple interventional strategies that activate mitophagy have demonstrated potential in ameliorating AD pathology and cognitive function. For instance, compounds such as urolithin A, NAD+ precursors, and rapamycin can promote mitophagy, enhance mitochondrial function, and alleviate neuronal damage and inflammatory responses in AD models (Pradeepkiran et al. 2022; Zheng et al. 2024). Additionally, natural products like ginsenoside Rg1 have been confirmed to improve AD‐related cognitive deficits by activating the PINK1‐Parkin‐mediated mitophagy pathway (N. Wang et al. 2023).

In summary, as a crucial mechanism of mitochondrial quality control, mitophagy maintains mitochondrial functional integrity and cellular energy metabolism balance by selectively clearing damaged mitochondria. In neurodegenerative diseases such as AD, impaired mitophagy leads to the accumulation of damaged mitochondria, thereby exacerbating neuronal injury (Picca et al. 2021; X. ‐L. Wang et al. 2021; Mary et al. 2023; Y. Zhao et al. 2020).

#### 2.5.2 Mitophagy‐Related Pathways and Their Molecular Mechanisms

Mitophagy is a selective autophagy process specifically responsible for clearing damaged or superfluous mitochondria, thereby maintaining the quality and quantity balance of cellular mitochondria. Against the backdrop of the close association between mitochondrial dysfunction and the pathogenesis of AD, abnormalities in mitophagy have become a focal point in AD research. The molecular mechanisms of mitophagy primarily encompass two major pathways: the classical PINK1/Parkin‐dependent pathway and receptor‐mediated pathways, both of which exhibit functional impairments in AD.

As mentioned earlier, the canonical PINK1/Parkin pathway represents the most extensively studied mechanism in mitophagy. PINK1 (a mitochondrial protein kinase) undergoes rapid degradation in healthy mitochondria. However, upon loss of mitochondrial membrane potential, PINK1 accumulates on the outer mitochondrial membrane, where it recruits and activates Parkin (an E3 ubiquitin ligase). Activated Parkin mediates the ubiquitination of mitochondrial surface proteins, recruiting autophagy adaptor proteins such as OPTN and NDP52, which connect to the autophagosome formation machinery to encapsulate and degrade damaged mitochondria. This pathway regulates the selective clearance of mitochondria and is crucial for neuronal energy metabolism and survival. Studies have found that in AD patients and models, the expression and function of both PINK1 and Parkin are suppressed, leading to the accumulation of damaged mitochondria, enhanced oxidative stress, and neuronal dysfunction (Mary et al. 2023; X. Li et al. 2021).

Another significant mitophagy pathway is the receptor‐mediated route, which involves specific receptor proteins on the outer mitochondrial membrane, such as BNIP3, BNIP3L (NIX), and FUNDC1. These receptors directly bind to the autophagy‐related protein LC3 through their LC3‐interacting region (LIR), thereby mediating the autophagic clearance of mitochondria. In AD, reduced expression of BNIP3L impairs the mitophagy process, and studies have shown that activating BNIP3L‐mediated mitophagy can ameliorate neuronal damage in AD models (Kakoty et al. 2025; Y. Li et al. 2021).

The process of mitophagy is regulated by multiple mechanisms, including protein post‐translational modifications, signaling pathway regulation, and cellular metabolic status. For instance, ROS signals generated from mitochondrial oxidative stress can activate the ATM‐CHK2 pathway within the DNA damage response network, promoting PINK1 accumulation and autophagosome formation, thereby enhancing mitophagy (Q. ‐Q. Guo et al. 2025). Furthermore, the AMPK pathway plays a key role in regulating mitophagy, particularly in neurons and cardiomyocytes, where it helps maintain mitochondrial dynamic balance by promoting mitochondrial fission and autophagosome formation (Seabright and Lai 2020; Ajoolabady et al. 2022).

In the pathological environment of AD, aberrantly phosphorylated Tau protein not only directly damages mitochondria but also exacerbates mitochondrial dysfunction by interfering with mitophagy pathways. Pathological Tau inhibits the PINK1/Parkin pathway, reducing the clearance of damaged mitochondria and leading to elevated mitochondrial ROS levels, which further promotes neuronal apoptosis and cognitive decline (Song et al. 2021; Mary et al. 2023). Additionally, Tau protein can disrupt receptor‐mediated mitophagy by impairing the expression and function of receptors such as BNIP3L and FUNDC1, creating a vicious cycle that perpetuates mitochondrial impairment (Mary et al. 2023).

In contrast to AD, where impaired PINK1/Parkin pathway function leads to defective clearance of damaged mitochondria, research on Huntington's disease (HD) has revealed a mechanism by which mutant proteins excessively drive mitochondrial fission. Studies have found that mutant huntingtin protein engages in aberrant interactions with the mitochondrial protein Drp1, enhancing its GTPase activity and resulting in excessive mitochondrial fragmentation and abnormal distribution. This pathological interaction is prominently present in affected brain regions of HD patients, such as the cortex and striatum, but is not observed in unaffected regions like the cerebellum. Furthermore, in HD patient brain tissue, expression levels of the fission genes Drp1 and Fis1 are increased, while levels of the fusion genes Mfn1, Mfn2, and Opa1 are decreased, leading to a disruption in the balance of mitochondrial dynamics. Regarding mitochondrial transport and synaptic damage, HD model studies have shown a significant reduction in mitochondrial motility, particularly with severe impairment of anterograde transport, preventing mitochondria from effectively reaching nerve terminals. This transport defect causes an abnormal accumulation of mitochondria in the neuronal soma, along with a reduced distribution within neurites and synaptic sites. Synapses are regions with high ATP demand, and this decreased mitochondrial presence at synaptic sites leads to reduced local ATP levels, subsequently triggering synaptic degeneration. Research confirms a significant reduction in the immunoreactivity of synaptic proteins such as synaptophysin and PSD95 in HD models, suggesting the involvement of mutant huntingtin in the process of synaptic degeneration. Therefore, while both HD and AD involve mitochondrial dysfunction, HD is more characterized by mutant proteins driving excessive mitochondrial fission via Drp1, which in turn disrupts axonal transport and energy distribution (Reddy and Shirendeb 2012).

Thus, as the core mechanism of mitochondrial quality control, both the canonical PINK1/Parkin‐mediated pathway and receptor‐mediated pathways of mitophagy are impaired in AD, leading to the accumulation of mitochondrial damage and neuronal injury. Furthermore, abnormal Tau protein exacerbates mitochondrial dysfunction through multiple mechanisms that disrupt mitophagy, creating a vicious cycle (Kakoty et al. 2025; Mary et al. 2023; X. Li et al. 2021; Onishi et al. 2021).

### 2.6 Therapeutic Strategies Targeting Mitochondria

#### 2.6.1 Protective Effects of Mitochondria‐Targeted Small Molecules

Given the central role of mitochondrial dysfunction in AD, mitochondria‐targeted therapeutic strategies have emerged as a research hotspot. Among these, small molecule compounds demonstrate immense potential. This section focuses on two molecules: SS‐31 and diethyl (3,4‐dihydroxyphenethylamino)(quinolin‐4‐yl)methylphosphonate (DDQ).

SS‐31 is a cell‐permeable tetrapeptide capable of targeting mitochondria. Its mechanism of action involves specific binding to cardiolipin, a unique phospholipid in the inner mitochondrial membrane crucial for maintaining mitochondrial cristae structure and the stability of electron transport chain supercomplexes. By binding to cardiolipin, SS‐31 stabilizes mitochondrial cristae structure, optimizes electron transport chain function, and thereby reduces ROS production. Studies by Reddy's laboratory further reveal that in diabetic mouse models, SS‐31 not only reduces hydrogen peroxide and lipid peroxidation levels in the liver but also restores mitochondrial function by modulating mitochondrial dynamics‐related proteins (decreasing Drp1 and Fis1, increasing Mfn1, Mfn2, and Opa1) and upregulating biogenesis genes (PGC‐1α, Nrf1, Nrf2, TFAM) (Bhatti et al. 2021). In Aβ‐treated neurons and primary neurons from APP transgenic mice, SS‐31 restores mitochondrial axonal transport, reduces mitochondrial fragmentation, and increases synaptic activity. Furthermore, the combined use of SS‐31 and the mitochondrial division inhibitor 1 (Mdivi1) shows synergistic protective effects, reducing Aβ levels and increasing mtDNA copy number and cell survival rates more effectively than individual treatments (Reddy et al. 2018).

DDQ is a novel mitochondria‐targeted small molecule developed by Reddy's laboratory. Pharmacokinetic studies indicate that DDQ has a half‐life of 20 h in serum and 12 h in the brain (Vijayan et al. 2021), demonstrating its ability to cross the blood–brain barrier (BBB) and maintain effective concentrations within the brain. In the APP transgenic mouse model (Tg2576), effective concentrations of DDQ can be detected in skeletal muscle, serum, and brain tissues, with the highest concentration observed in skeletal muscle. Behavioral tests reveal that DDQ treatment significantly improves the performance of APP mice in the Morris water maze test, shortening the latency to find the platform (Bhatti et al. 2021) and increasing the time spent in the target quadrant (Vijayan et al. 2021). Mechanistic studies show that DDQ treatment decreases the expression of fission genes (Drp1, Fis1) and increases the expression of fusion genes (Mfn1, Mfn2, Opa1) and biogenesis genes (PGC1α, Nrf1, Nrf2, TFAM). Additionally, DDQ upregulates the expression of autophagy/mitophagy‐related genes (such as ATG5, Beclin1, PINK1) and longevity genes (Sirtuins). Transmission electron microscopy confirms that DDQ treatment reduces fragmented mitochondria and significantly increases mitochondrial length in the hippocampus and cortical tissues of APP mice. Golgi staining further indicates that DDQ treatment significantly increases the number and length of dendritic spines in hippocampal neurons of APP mice. These findings suggest that DDQ exerts neuroprotective effects in AD models through multiple pathways, including antioxidant activity, regulation of mitochondrial dynamics, and autophagy (Vijayan et al. 2021).

#### 2.6.2 Other Potential Intervention Strategies

In addition to the small molecules mentioned above, several other strategies targeting mitochondrial function merit attention.

Mdivi‐1 is a selective inhibitor of the mitochondrial fission protein DRP1. It blocks DRP1 self‐assembly and GTPase activity, thereby significantly inhibiting excessive mitochondrial fragmentation (Johri 2021). Mdivi‐1 has demonstrated robust neuroprotective effects across various AD models (Baek et al. 2017; Reddy et al. 2017; Manczak et al. 2019), including reduced Aβ deposition, decreased BACE1 expression, attenuated oxidative stress and lipid peroxidation, and restoration of synaptic function with improved cognitive performance. Recent studies also indicate that Mdivi‐1 can mitigate Aβ‐induced mitochondrial fragmentation and rescue synaptic transmission deficits (B. Zhao et al. 2024).

Beyond small molecules, peptide‐based interventions have emerged as promising candidates. P110 is a heptapeptide designed based on the homologous region of DRP1's interaction with its outer membrane receptor FIS1 (Austad et al. 2022; Atlante et al. 2022). By specifically blocking DRP1 activation, P110 reduces excessive mitochondrial fission and has been shown to alleviate cell death and mitochondrial dysfunction in AD models (Macdonald et al. 2018).

### 2.7 The Role of Mitochondria and the Gut–Brain Axis in AD

#### 2.7.1 Mutual Interactions Between the Gut Microbiota and Mitochondrial Function

**FIGURE 3:**
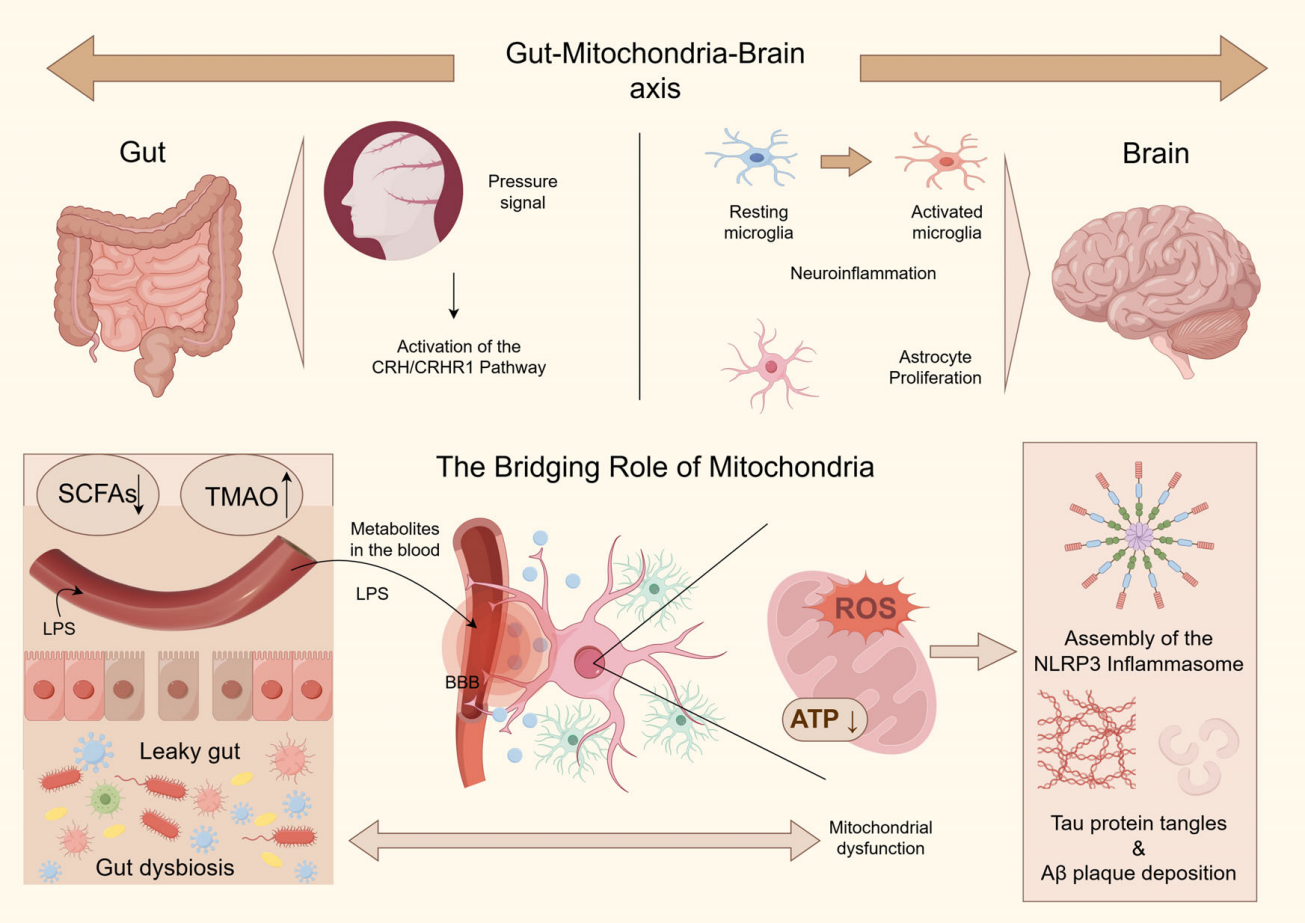
Schematic diagram of the interaction mechanism between gut–brain axis‐mediated mitochondrial dysfunction and neuroinflammation in AD. Gut microbiota dysbiosis leads to a decrease in beneficial metabolites (such as short‐chain fatty acids, SCFAs) and an increase in harmful substances like lipopolysaccharides (LPS), compromising intestinal barrier integrity and allowing these substances to enter the brain via the circulatory system (Kumar et al. 2025). These gut‐derived signaling molecules can directly affect mitochondrial function in neurons and glial cells, inducing energy metabolism disorders, accumulation of ROS, and impaired mitophagy (Ajith and Sreejith 2026). Mitochondrial dysfunction further activates the NLRP3 inflammasome in microglia and astrocytes, promoting the release of pro‐inflammatory factors such as IL‐1β, exacerbating neuroinflammatory responses, and further facilitating Aβ deposition and Tau pathology (Kulkarni et al. 2025; C. He et al. 2025). Simultaneously, the inflammatory state in the central nervous system can reciprocally influence the intestinal environment through neuroendocrine and immune feedback pathways, forming a bidirectional “brain–gut” vicious cycle (Shaikh et al. 2023; Kasarello et al. 2023). The Fig 3 was created by Figdraw. Copyright Code: YOAUI000a5.

There exists a complex and intimate interplay between the gut microbiota and mitochondrial function, which collectively influences host energy metabolism, inflammatory status, and cellular homeostasis through multiple pathways, thereby exerting a profound impact on brain function and the development and progression of neurodegenerative diseases. A schematic overview of the bidirectional interactions between the gut–brain axis and mitochondrial dysfunction in AD is presented in Figure 3.

Primarily, the gut microbiota directly regulates mitochondrial energy production, oxidative phosphorylation, calcium ion homeostasis, and oxidative stress responses through its metabolites, including short‐chain fatty acids (SCFAs), secondary bile acids, tryptophan metabolites, and trimethylamine N‐oxide (TMAO). These metabolites not only serve as mitochondrial energy substrates or signaling molecules but also maintain the dynamic balance of mitochondrial function by modulating mitochondrial biogenesis and autophagy. For instance, SCFAs can promote mitochondrial biogenesis and enhance antioxidant capacity, thereby mitigating neuroinflammation and oxidative damage in the nervous system (Qiao et al. 2024; Yadav et al. 2022).

Furthermore, dysbiosis of the gut microbiota is frequently accompanied by mitochondrial dysfunction, characterized by reduced activity of mitochondrial respiratory chain complexes, decreased ATP synthesis, and increased ROS generation. This pathological state is particularly prominent in AD and other neurodegenerative disorders (L. Liu et al. 2021; Madamanchi et al. 2025).

On the other hand, mitochondria, as metabolic hubs of the cell, not only regulate cellular energy metabolism but are also involved in the self‐renewal, apoptosis, and barrier function maintenance of intestinal epithelial cells. Optimal mitochondrial function in intestinal epithelial cells is crucial for preserving intestinal barrier integrity, modulating immune responses, and preventing the invasion of harmful substances and pathogens. For example, under the regulation of the gut microbiota, enteroendocrine cells (EECs) undergo significant changes in mitochondrial function and morphology, accompanied by the modulation of cellular calcium signaling—a process termed “EEC awakening,” which plays an important role in nutrient sensing and gut–brain axis signaling (Alsudayri et al. Alsudayri et al. 2024). Additionally, mitochondrial dysfunction in intestinal epithelial cells can lead to inadequate energy supply and heightened oxidative stress, promoting intestinal inflammation and increased barrier permeability, which in turn exacerbates gut microbiota dysbiosis, forming a vicious cycle (Alula et al. 2023; Smith et al. 2021).

Numerous studies have further revealed that the gut microbiota modulates mitochondrial functional status by influencing host metabolites and signaling pathways, thereby affecting systemic inflammation, metabolic diseases, and the onset of neurological disorders. For example, the amino acid metabolite aspartate exerts antioxidant and cytoprotective effects by regulating gut microbiota composition and RIP‐dependent mitochondrial function (Jin et al. 2025). Meanwhile, neuroinflammation resulting from gut microbiota dysbiosis is closely associated with impaired mitochondrial function, serving as a critical pathogenic link in depression and Parkinson's disease (H. Zhao et al. 2025; Liang et al. 2021). Furthermore, the gut microbiota can influence mitochondrial biogenesis and antioxidant capacity by modulating mitochondrial‐associated proteins such as sirtuins, thereby participating in the regulation of neurodegenerative diseases (Munteanu et al. 2024).

Based on the aforementioned content, it is evident that the mutual influence between the gut microbiota and mitochondrial function constitutes a bidirectional regulatory network: the gut microbiota and its metabolites modulate mitochondrial energy metabolism and antioxidant mechanisms, thereby affecting cerebral energy supply and inflammatory status; conversely, mitochondrial functional status influences the barrier function of intestinal epithelial cells and the stability of the microecological environment, maintaining intestinal homeostasis. Intervention strategies targeting this axis—such as the use of probiotics, dietary modifications, and drugs targeting mitochondrial function—hold promise as potential therapeutic approaches for a range of conditions, including neurodegenerative and metabolic diseases (Wen et al. 2025; Rukavina Mikusic et al. 2025).

#### 2.7.2 Pathological Correlation Between Gut–Brain Axis Imbalance and AD

The microbiota–gut–brain axis refers to a bidirectional communication system between the gut microbiota and the central nervous system, mediated through neural, immune, endocrine, and metabolic pathways. In recent years, growing evidence has revealed that dysregulation of this axis plays a critical role in the pathogenesis of AD. Gut microbiota dysbiosis not only disrupts digestive system homeostasis but also impairs intestinal barrier function, leading to increased intestinal permeability. This heightened permeability allows bacteria and their products, such as lipopolysaccharide (LPS), to enter the systemic circulation, activating immune responses both peripherally and within the brain. This process induces neuroinflammation and promotes the progression of AD‐related pathology (Giridharan et al. 2022; Dhanawat et al. 2025).

Specifically, intestinal inflammation and gut dysbiosis affect the central nervous system through multiple pathways. First, microbial metabolites such as SCFAs, neurotransmitters, and neuroactive molecules can directly or indirectly modulate neural function and immune responses. For example, SCFAs influence neuronal health by regulating inflammatory responses and maintaining BBB integrity (Ajith and Sreejith 2026; L. et al. 2023). Second, gut microbiota abnormalities can activate the NLRP3 inflammasome in both the gut and brain, promoting the release of pro‐inflammatory cytokines such as IL‐1β, IL‐6, and TNF‐α, thereby inducing neuroinflammation and neuronal damage (Mo et al. 2024; J. He et al. 2024). Additionally, gut dysbiosis may increase the permeability of both intestinal and brain barriers, triggering systemic inflammatory responses that promote abnormal accumulation of amyloid and Tau proteins, ultimately accelerating the progression of AD pathology (Goyal et al. 2021; Bhattacharya et al. 2025).

Both animal studies and clinical research support the role of the gut–brain axis in the pathogenesis of AD. AD patients and model animals consistently demonstrate reduced gut microbiota diversity, decreased beneficial bacteria such as “Lactobacillus,” and increased proportions of pathogenic bacteria, accompanied by impaired intestinal barrier function and enhanced inflammatory responses (Cho et al. 2021; Yin et al. 2025). Interventions modulating the gut microbiota, including probiotic supplementation and fecal microbiota transplantation, have been shown to improve cognitive function, alleviate neuroinflammation and oxidative stress, and delay AD pathological progression (Y. Wang and Dykes 2022; Marizzoni et al. 2025; Ren et al. 2025). For example, traditional Chinese medicine formulations like Danggui Shaoyao San ameliorate AD‐related pathology and cognitive impairment by regulating gut microbiota composition and metabolites, thereby reducing the expression of pro‐inflammatory factors in the brain (J. He et al. 2024). Additionally, dietary interventions aimed at modulating microbial metabolites, such as SCFAs and neurotransmitters, are considered potential therapeutic strategies (Frausto et al. 2021).

In summary, intestinal inflammation and gut microbiota dysbiosis activate immune responses in the central nervous system through mitochondria‐mediated inflammatory signaling pathways, thereby promoting AD‐associated neuroinflammation and neuronal damage. The bidirectional communication of the gut–brain axis enables alterations in the gut microenvironment to influence cerebral pathological states, establishing it as a significant component of AD pathogenesis. Therapeutic strategies targeting the gut–brain axis—such as probiotics, prebiotics, fecal microbiota transplantation, and modulation of microbial metabolites—hold promise as novel approaches for the prevention and treatment of AD (Goyal et al. 2021; Marizzoni et al. 2025; Y. Wang 2023).

#### 2.7.3 The Potential of Mitochondria in Interventions for the Gut–Brain Axis

Modulating mitochondrial function to improve intestinal barrier integrity and mitigate neuroinflammation has emerged as an innovative approach for gut–brain axis interventions in AD. As the central hub of cellular energy metabolism, mitochondria not only sustain neuronal energy demands but also participate in numerous biological processes including calcium homeostasis, oxidative stress responses, and apoptosis, making them crucial for nervous system health. Recent research demonstrates that the gut microbiota influences cerebral mitochondrial function through its metabolites, thereby regulating neurological health status. Microbial metabolites such as SCFAs, secondary bile acids, and tryptophan derivatives can cross the BBB to modulate mitochondrial energy production, calcium regulation, mitophagy, and oxidative stress levels in neurons and glial cells, highlighting mitochondria's pivotal intermediary role in the microbiota–gut–brain axis (Qiao et al. 2024).

Research on stress‐related gut–brain axis disruption mechanisms has revealed that psychological stress activates corticotropin‐releasing hormone (CRH) and its receptor CRHR1, damaging colonic epithelial cell mitochondria and leading to gut microbiota dysbiosis. This subsequently compromises intestinal barrier function and triggers systemic and central nervous system inflammatory responses. The CRH‐CRHR1‐mitochondrial signaling axis provides a potential therapeutic target for intestinal barrier integrity and neuroinflammation, as targeting this pathway may ameliorate stress‐induced gut microbial imbalance and inflammatory responses (Y. Zhang et al. 2024). Furthermore, mitochondrial dysfunction has been confirmed as a critical pathological component in multiple neurodegenerative diseases, with gut microbiota participating in the onset and progression of these disorders by regulating mitochondrial bioenergetics and dynamic balance (Ju et al. 2025).

In AD, gut microbiota dysbiosis contributes to neuroinflammation, oxidative stress, and mitochondrial dysfunction. Restoring gut microbial balance through interventions such as probiotics, prebiotics, and their metabolites (e.g., postbiotics) can help repair intestinal barrier function, reduce the release of pro‐inflammatory factors, and protect mitochondrial function in neural cells, thereby slowing AD progression. Relevant studies indicate that probiotics and gut microbiota modulators can promote the production of SCFAs, maintain BBB integrity, suppress neuroinflammation, and improve cognitive function (Kulkarni et al. 2025; Aran et al. 2025). For instance, nicotinamide mononucleotide (NMN), a precursor for NAD+ synthesis, has been shown to ameliorate gut microbiota composition in AD mouse models, promote the proliferation of SCFA‐producing bacteria, restore intestinal barrier function, and alleviate cognitive impairment. These findings demonstrate the therapeutic potential of concurrently modulating mitochondrial function and gut microbiota (X. Zhao et al. 2023).

Furthermore, enhancing mitochondrial function can be achieved by reducing ROS generation, maintaining mitochondrial membrane potential, and promoting mitophagy, thereby protecting both intestinal epithelial cells and neurons from damage. For example, the traditional Chinese medicine component Radix Hedysari polysaccharides (RHP) has demonstrated cognitive‐improving effects in AD models by modulating the gut–brain axis and providing mitochondrial protection, suggesting the clinical translational potential of strategies that preserve intestinal barrier integrity through mitochondrial function regulation (S. Yang et al. 2025).

In summary, mitochondria play a pivotal regulatory role within the gut–brain axis, where gut microbiota and their metabolites influence mitochondrial function to modulate intestinal barrier integrity and neuroinflammatory status. Therapeutic strategies targeting mitochondrial dysfunction can not only improve the gut microenvironment but also reduce inflammatory and oxidative damage in the nervous system, opening new avenues for treating neurodegenerative diseases like AD. Precise interventions targeting this axis will contribute to achieving early prevention and treatment of AD (Qiao et al. 2024; Y. Zhang et al. 2024; Ju et al. 2025).

### 2.8 Future Research Directions and Prospects

AD, as a complex neurodegenerative disorder, involves multiple pathological pathways in its pathogenesis. Among these, mitochondrial dysfunction has garnered significant attention in recent years as a core factor in AD development. Future research should focus on elucidating the specific mechanisms of mitochondrial dysfunction in AD, developing early diagnostic biomarkers, and advancing mitochondria‐based therapeutic strategies, with the goal of providing more precise and effective approaches for AD prevention and treatment.

First and foremost, elucidating the multifaceted mechanisms of mitochondrial dysfunction in the pathogenesis of AD represents a critical focus for future research. Substantial evidence indicates that impairments in mitochondrial energy metabolism, oxidative stress, disruption of calcium homeostasis, dysregulated mitochondrial dynamics (including aberrant fusion and fission), and defective mitophagy are all implicated in the onset and progression of AD. For instance, dysfunction of the mitochondrial respiratory chain complexes, particularly Complex IV, is closely associated with neuropathology in AD brain regions and exhibits a negative correlation with amyloid‐β (Aβ) plaque burden (Tian et al. 2025). Furthermore, mitochondrial‐associated proteins such as VDAC1 and BNIP3L/NIX, as well as genes involved in mitochondrial‐immune interactions (e.g., TSPO, HIGD1A), have been linked to AD pathology and cognitive deficits, underscoring the importance of investigating the crosstalk between mitochondrial dysfunction and neuroinflammation in future studies (Kakoty et al. 2025; Argueti‐Ostrovsky et al. 2025; Meng et al. 2025). Consequently, utilizing multi‐omics approaches—particularly mitochondrial omics, proteomics, and miRNA‐Omics—to systematically delineate the molecular networks linking mitochondrial dysfunction to AD pathology will be essential for advancing our understanding (Liao et al. 2024; Rivera et al. 2023).

Second, developing early diagnostic biomarkers related to mitochondrial dysfunction is crucial for the early identification and intervention of AD. Current research has found that mitochondrial DNA oxidative damage markers, such as 8‐oxoguanine (8‐oxoG), are significantly elevated in specific populations and may correlate with cognitive function, offering potential indicators for precise diagnosis (Reid et al. 2022). Furthermore, alterations in the expression of mitochondrial respiratory chain‐related genes and their interactions with immune responses also hold promise as biomarkers for both diagnosis and monitoring disease progression (Meng et al. 2025). Therefore, future efforts should strengthen multicenter, large‐scale clinical studies, integrating functional neuroimaging and liquid biopsy technologies to advance the clinical validation and application of these biomarkers.

Furthermore, therapeutic strategies targeting mitochondrial dysfunction represent a pivotal direction for future AD research. Given the limited efficacy of conventional therapies directed at Aβ and Tau proteins, interventions focusing on restoring mitochondrial bioenergetics, mitigating oxidative stress, regulating mitochondrial dynamics, and promoting mitophagy have become major research priorities (S. Wang et al. 2025; Gulcan and Orhan 2021). The application of nanotechnology in mitochondrial‐targeted drug delivery shows considerable promise by enhancing drug stability and targeting precision, thereby strengthening neuroprotective efficacy (Ergin 2025). Moreover, emerging mitochondrial gene‐editing technologies, such as zinc‐finger nucleases and base editors, provide novel tools for modulating mitochondrial genetic abnormalities and hold potential for future gene therapy in AD (Hong et al. 2025). Exercise interventions have been demonstrated to improve mitochondrial function by modulating the expression of mitochondrial fusion protein Mfn2, thereby alleviating AD‐related pathology and cognitive deficits (H. Li 2025). Simultaneously, natural compounds such as flavonoids and phytochemicals exhibit potential therapeutic value via their regulatory effects on mitochondrial function (Gulcan and Orhan 2021; Rahman et al. 2021). Future research should integrate basic and clinical studies to optimize treatment regimens and systematically evaluate their efficacy and safety.

Finally, mitochondrial dysfunction in microglia can activate the NLRP3 inflammasome, thereby promoting neuroinflammatory responses and accelerating the pathological progression of AD (L. Zhang et al. 2024; Z. Li et al. 2024). Future research should further elucidate mitochondrial‐immune signaling pathways to explore their potential as therapeutic targets, while also investigating the roles of mitochondrial trafficking and intercellular mitochondrial transfer in neuroprotection (Wei et al. 2024; Javadpour et al. 2024).

With the intensification of societal aging, the relationship between metabolic diseases such as diabetes and AD has become increasingly evident. Mitochondrial dysfunction plays a critical role in diabetes‐associated cognitive impairment, making the regulation of oxidative stress and mitophagy potential therapeutic targets (Koshatwar et al. 2023; C. Wang et al. 2025). Future efforts should strengthen multidisciplinary integrated research to consolidate the mechanisms linking metabolic and neurodegenerative diseases, thereby advancing the development of personalized therapeutic approaches.

## 3 Conclusion

AD, as a complex neurodegenerative disorder, involves multiple pathological processes in its pathogenesis, among which mitochondrial dysfunction has been widely recognized as a core component. Mitochondria are not only the central hub for cellular energy metabolism but also play critical roles in maintaining neuronal homeostasis, regulating oxidative stress responses, and participating in apoptotic pathways.

This article delves into the multifaceted nature of mitochondrial dysfunction, ranging from classical mitochondrial energy metabolism deficits and oxidative stress to the imbalance in the intricate regulation of mitochondrial dynamics (fusion and fission), and further to defects in mitophagy, a key quality control mechanism. We emphasize how Aβ and p‐Tau directly drive mitochondrial fragmentation and dysfunction through aberrant interactions with key mitochondrial proteins, Drp1 and VDAC1. Furthermore, dysfunction of mitochondria‐associated membranes (MAMs), the hubs connecting mitochondria and the ER, reveals the interconnection of signaling pathways between mitochondria and other organelles, deepening our understanding of novel mechanisms in AD. Simultaneously, the impact of the gut–brain axis on mitochondrial function highlights the multifactorial complexity of AD pathology, suggesting that future research must integrate the interplay of genetic and environmental factors.

On the therapeutic front, although findings regarding interventions targeting specific molecular mechanisms and their effects have shown variability, the overall consensus indicates that therapeutic strategies targeting mitochondria hold significant promise. Mitochondria‐targeted small molecules, exemplified by SS‐31 and DDQ, have demonstrated neuroprotective effects and improved cognitive function in AD models through various mechanisms, including stabilizing mitochondrial membrane structure, scavenging free radicals, regulating mitochondrial dynamics, and enhancing mitophagy. These multidimensional interventions reflect a paradigm shift in AD therapeutic research from single targets toward multi‐target, multi‐mechanism synergistic regulation.

Future research should prioritize elucidating the precise molecular mechanisms underlying mitochondrial dysfunction, particularly the dynamic processes of interaction between Aβ/p‐Tau and mitochondrial proteins. Simultaneously, integrating modern high‐throughput omics technologies with precision medicine principles is crucial to accelerate the discovery of biomarkers associated with mitochondrial dysfunction, aiming to facilitate early diagnosis of AD. Moreover, clinical translational research must intensify the evaluation of the safety and efficacy of existing and emerging mitochondria‐targeted strategies and explore personalized treatment approaches to achieve the goals of precision medicine. In summary, deepening our understanding of the central role of mitochondrial dysfunction in AD and integrating findings from diverse research perspectives will provide novel theoretical foundations and therapeutic avenues to combat this devastating disease, ultimately driving transformative breakthroughs in the field of neurodegenerative disease therapeutics.

## Author Contributions

**Tianyi Gu**, as the first author and submitting author, contributed to writing – original draft, conceptualization, methodology, investigation, and visualization, critically revising the manuscript. In accordance with the authorship order, **Hangyan Guo** and **Zixin Guo** jointly contributed to the investigation, primarily responsible for the literature search and data collection. **Shengyu Hua**, serving as the corresponding author, contributed to writing – review and editing, with a focus on critical review of the manuscript, in addition to providing critical guidance and oversight throughout the work.

## Funding

The authors have nothing to report.

## Conflicts of Interest

The authors declare no conflicts of interest.

## Data Availability

Data sharing is not applicable to this article as no datasets were generated or analyzed during the current study.
